# NT-proBNP ratio is a potential predictor for COVID-19 outcomes in adult Chinese patients: a retrospective study

**DOI:** 10.1038/s41598-024-56329-2

**Published:** 2024-03-11

**Authors:** Dan Li, Wu He, Bo Yu, Dao Wen Wang, Li Ni

**Affiliations:** Division of Cardiology, Department of Internal Medicine and Hubei Key Laboratory of Genetics and Molecular Mechanisms of Cardiological Disorders, Tongji Hospital, Tongji Medical College, Huazhong University of Science and Technology, 1095# Jiefang Ave., Wuhan, 430030 China

**Keywords:** NT-proBNP ratio, In-hospital mortality, COVID-19, Time-dependent ROC curve, Infectious diseases, Risk factors

## Abstract

Despite the progressive decline in the virulence of the novel coronavirus, there has been no corresponding reduction in its associated hospital mortality. Our aim was to redefine an accurate predictor of mortality risk in COVID-19 patients, enabling effective management and resource allocation. We conducted a retrospective analysis of 2917 adult Chinese patients diagnosed with COVID-19 who were admitted to our hospital during two waves of epidemics, involving the Beta and Omicron variants. Upon admission, NT-proBNP levels were measured, and we collected demographic, clinical, and laboratory data. We introduced a new concept called the NT-proBNP ratio, which measures the NT-proBNP level relative to age-specific maximum normal values. The primary outcome was all-cause in-hospital mortality. Our analysis revealed a higher in-hospital mortality rate in 2022, as shown by the Kaplan–Meier Survival Curve. To assess the predictive value of the NT-proBNP ratio, we employed the time-dependent receiver operating characteristic (ROC) curve. Notably, the NT-proBNP ratio emerged as the strongest predictor of mortality in adult Chinese hospitalized COVID-19 patients (area under the curve, AUC = 0.826; adjusted hazard ratio [HR], 3.959; 95% confidence interval [CI] 3.001–5.221; P < 0.001). This finding consistently held true for both the 2020 and 2022 subgroups. The NT-proBNP ratio demonstrates potential predictive capability compared to several established risk factors, including NT-proBNP, hsCRP, and neutrophil-to-lymphocyte ratio, when it comes to forecasting in-hospital mortality among adult Chinese patients with COVID-19.

**Trial registration** Clinical Trial Registration: www.clinicaltrials.gov NCT05615792.

## Introduction

Cardiovascular complications are common following infection with severe acute respiratory syndrome coronavirus 2 (SARS-CoV-2), and they significantly contribute to the morbidity and mortality associated with the novel coronavirus disease (COVID-19)^1^. N-terminal pro B-type natriuretic peptide (NT-proBNP) is a marker that indicates cardiac stress related to hemodynamic changes, and it has shown promise in predicting the risk of complications such as heart failure (HF) and pulmonary embolism in COVID-19 patients^2,3^.

A retrospective study conducted in China revealed that NT-proBNP levels were frequently elevated in COVID-19 patients. Furthermore, these elevated levels were strongly and independently correlated with mortality even after adjusting for relevant confounding factors, including chronic HF and acute HF^4^. However, a prospective study conducted in Norway reported different results, stating that NT-proBNP levels did not show a significant association with mortality after adjusting for various factors such as demographics, cardiovascular disease, body mass index, estimated glomerular filtration rate, symptom duration, and National Early Warning Score (NEWS)^5^.

The estimation of prognosis for COVID-19 based solely on the absolute value of NT-proBNP is inaccurate due to variations in the normal reference range across different age groups. To address this limitation, we propose the concept of the NT-proBNP ratio, which considers NT-proBNP levels relative to age-specific maximum values and takes into account changes in the normal reference range among different age groups.

Since the outbreak of the novel coronavirus in Wuhan, China experienced two significant waves of epidemics, which were associated with the Beta and Omicron variants, mainly occurring in spring 2020 and from winter 2022 to spring 2023, respectively. The objective of our study is to investigate the potential value of the NT-proBNP ratio and its role in risk stratification among adult Chinese patients admitted to the hospital with COVID-19.

## Methods

### Participants and data collection

We included consecutive adult Chinese patients (> 18 years) who were admitted to Tongji Hospital during two different periods: February 1, 2020, to April 26, 2020 (Beta variant), and December 1, 2022, to February 28, 2023 (Omicron variant). All enrolled patients had confirmed SARS-CoV-2 infection. Confirmation of SARS-CoV-2 infection required two consecutive positive results from high-throughput sequencing or real-time reverse transcriptase-polymerase chain reaction assays of nasal and pharyngeal swabs. Additionally, cases with a clinical diagnosis considered imaging evidence of pneumonia, along with relevant epidemiological background and clinical symptoms, were also included. The enrollment criteria for patients in 2020 included men or non-pregnant women aged 18 years or older, who were diagnosed with either confirmed or clinically diagnosed cases according to the diagnosis and treatment of COVID-19 guidelines (sixth version) published by the National Health Commission of China (NHCC). The patients were classified into mild, moderate, severe, and critical types based on the disease severity. The classification was also based on the diagnosis and treatment of COVID-19 guidelines (sixth version by NHCC). Attending physicians and study physicians reviewed medical histories and examinations to collect all relevant data. The investigation adheres to the principles outlined in the Declaration of Helsinki (Br Med J 1964; ii: 177). Patients without available NT-proBNP data were excluded from the study.

Data pertaining to demographics, primary comorbidities (including hypertension, diabetes, coronary heart disease, chronic kidney disease, and tumors), physical examinations, routine blood tests, blood chemistry, coagulation function, markers of cardiac function, and diabetes-related indices were extracted from electronic medical records. The physical examination findings and the initial laboratory results of the aforementioned indicators upon admission (within 24 to 48 h) were recorded. The test results were mainly checked by double-antibody sandwich method of chemiluminescence technique based on Roche platform. The Heart Failure Association of the European Society of Cardiology (ESC) has provided recommended cutoff values for normal NT-proBNP levels in different age groups: > 450 pg/mL for patients under 50 years, > 900 pg/mL for patients between 50 and 75 years, and > 1800 pg/mL for patients aged 75 years or older^6^.

This retrospective observational registry was conducted in adherence to the guidelines and the study were approved by the Ethics Commission of Tongji Hospital, Tongji Medical College, Huazhong University of Science and Technology, located in Wuhan, Hubei Province, China (Permission No. TJ-IRB20200229 and No. TJ-IRB20210138). Informed consent was obtained from all subjects and/or their legal guardians.

### Outcome and statistical analysis

The primary endpoint of this cohort study was in-hospital all-cause mortality. Patients were followed from admission until the observation data points (February 1, 2020, to April 26, 2020, and December 1, 2022, to February 28, 2023) to assess the risk of in-hospital mortality. Attending physicians and study physicians thoroughly evaluated the medical records to gather the necessary follow-up data.

The normality of distribution and homoscedasticity of each dataset were evaluated using D'Agostino's and Pearson's omnibus tests. For normally distributed continuous data, the mean values along with their standard deviations were presented and compared using Student's t-test. The Wilcoxon rank-sum test was employed to compare continuous non-normally distributed data, where the median values and interquartile ranges (IQR) were reported. If applicable, categorical data, expressed as counts (percentages), were compared using the Chi-square test, Fisher's exact test, and Cochran–Mantel–Haenszel test. Bonferroni's correction was used to evaluate the statistical significance of multiple comparisons.

The binary cutoff value was selected based on the maximum Youden index. Survival analysis utilized Kaplan–Meier curves and Cox proportional hazard models to compare the incidence of endpoint events among both the overall patient population and different subgroups. The time-dependent receiver operating characteristic (ROC) curve is used to explore the optimal predictor for in-hospital mortality of COVID-19. Statistical analysis and data visualization were conducted using GraphPad Prism (version 9.0; San Diego, CA, USA), R (version 4.1.1; R Foundation for Statistical Computing, Vienna, Austria), and SPSS (version 24.0; IBM, Armonk, NY, USA). The main R packages used include "survival", "survminer", "pROC" and "timeROC". The threshold for statistical significance was set at P < 0.05.

## Results

### Demographics and baseline characteristics

**Figure 1:**
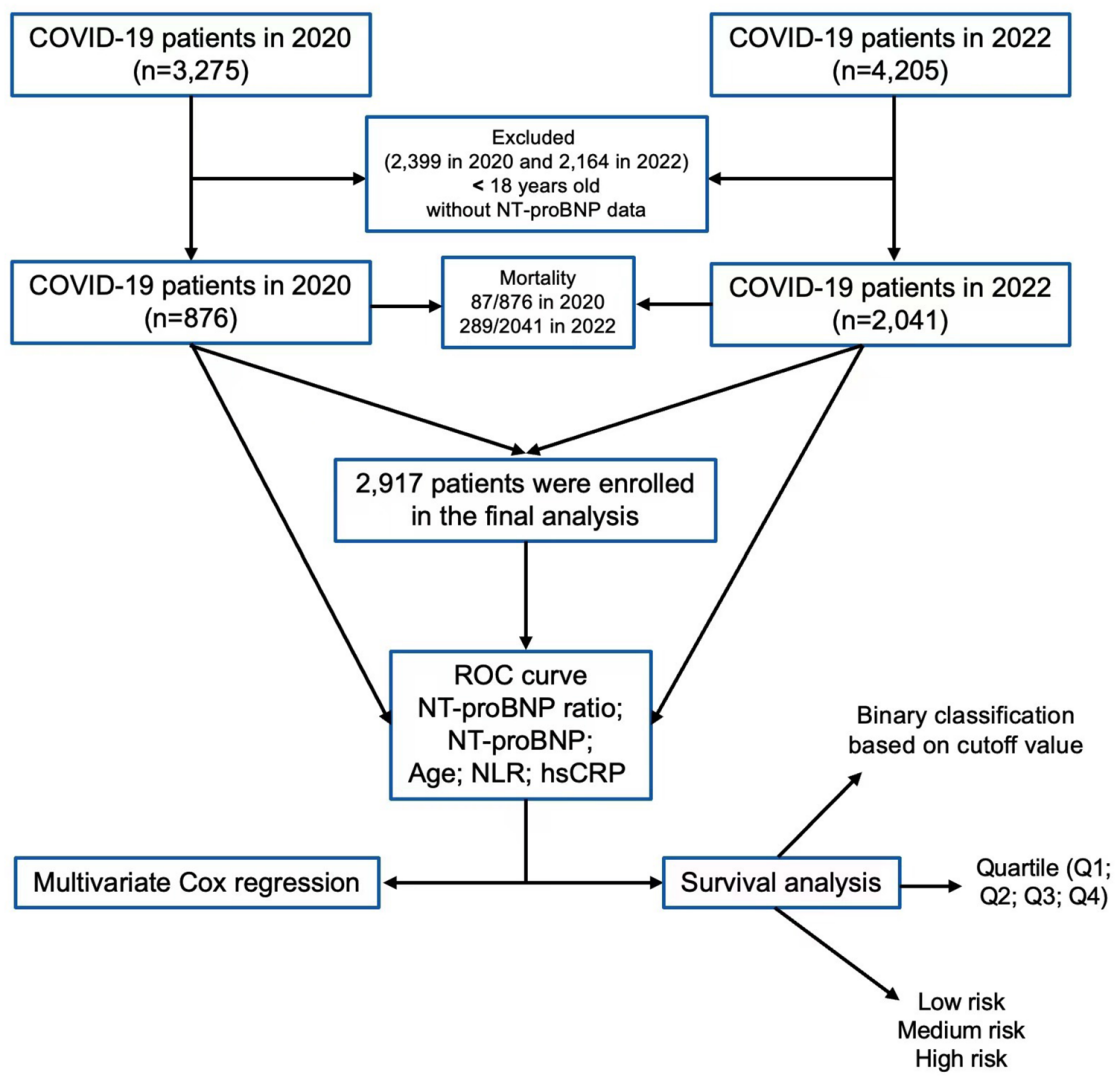
The flowchart of study design.

Between February 1, 2020, and April 26, 2020, Tongji Hospital diagnosed a total of 3275 consecutive individuals with COVID-19 (Fig. 1). Among them, 876 (26.7%) underwent measurements of NT-proBNP, neutrophil-to-lymphocyte ratio (NLR), and hsCRP. The median values at the initial assessment were as follows: NT-proBNP (123 pg/mL, IQR 39–384), NT-proBNP ratio (0.13, IQR 0.05–0.38), NLR (3.25, IQR 2.01–6.21), and hsCRP (14.1 mg/L, IQR 2.2–59.6) (Fig. 1, Table 1).

**Table 1:** Baseline data of COVID-19 patients in 2020 and 2022.

	Total COVID-19 patients (n = 2917)	COVID-19 patients in 2020 (n = 876)	COVID-19 patients in 2022 (n = 2041)
Gender: male (%)	1714 (58.8)	448 (51.1)	1266 (62.0)
Age, years	68 (58–77)	63 (53–71)	70 (60–79)
Hospitalization time (days)	12 (8–19)	20 (12–30)	10 (7–15)
Severity (%)			
Mild	1191 (40.8)	371 (42.4)	820 (40.2)
Moderate	988 (33.9)	312 (35.6)	676 (33.1)
Severe	618 (21.2)	163 (18.6)	455 (22.3)
Critical	120 (4.1)	30 (3.4)	90 (4.4)
Vital signs			
Systolic blood pressure (mmHg)	127 (117–141)	130 (118–144)	126 (116–140)
Diastolic blood pressure (mmHg)	78 (70–86)	80 (72–89)	77 (70–85)
Respiratory rate (times per minute)	20 (20–20)	20 (20–24)	20 (20–20)
Pulse rate (times per minute)	82 (76–95)	89 (79–101)	80 (75–91)
Temperature (°C)	36.5 (36.3–36.8)	36.5 (36.3–37.0)	36.5 (36.3–36.8)
Original comorbidities (%)			
Hypertension (%)	1023 (35.1)	271 (30.9)	752 (36.8)
Diabetes (%)	542 (18.6)	126 (14.4)	416 (20.4)
Coronary heart disease (%)	360 (12.3)	64 (7.3)	296 (14.5)
CKD (%)	376 (12.9)	7 (0.8)	369 (18.1)
Tumor (%)	272 (9.3)	19 (2.2)	253 (12.4)
Laboratory results			
Routine blood test			
Red blood cell count, × 10^12^/L	4.03 (3.57–4.45)	4.13 (3.72–4.50)	3.97 (3.48–4.43)
White cell count, × 10^9^/L	6.22 (4.62–8.76)	5.90 (4.64–7.94)	6.42 (4.60–9.12)
Neutrophil count, × 10^9^/L	4.53 (2.97–7.04)	3.86 (2.79–5.81)	4.81 (3.12–7.55)
Lymphocyte count, × 10^9^/L	0.90 (0.57–1.36)	1.12 (0.75–1.60)	0.81 (0.51–1.22)
Neutrophil–lymphocyte ratio	4.81 (2.56–10.41)	3.25 (2.01–6.21)	5.72 (2.94–12.63)
Monocyte count, × 10^9^/L	0.49 (0.35–0.68)	0.49 (0.37–0.64)	0.49 (0.33–0.70)
Alanine aminotransferase, U/L	21 (14–35)	22 (15–36)	21 (14–34)
Aspartate aminotransferase, U/L	27 (19–40)	26 (19–36)	27 (19–42)
Lactate dehydrogenase, U/L	259 (205–361)	262 (209–366)	257 (204–360)
Total bilirubin, μmol/L	8.8 (6.3–12.6)	9.1 (6.7–13.0)	8.5 (6.1–12.4)
Total protein, g/L	66.8 (62.6–70.9)	68.6 (64.9–72.3)	66.1 (61.7–70.2)
Blood biochemistry			
Albumin-globulin ratio	1.07 (0.89–1.29)	1.10 (0.91–1.32)	1.06 (0.88–1.28)
Alkaline phosphatase, U/L	69 (57–88)	66 (55–84)	71 (57–90)
Total cholesterol, mmol/L	3.65 (3.05–4.37)	3.75 (3.18–4.42)	3.61 (2.98–4.34)
Triglyceride, mmol/L	1.33 (0.98–1.86)	1.23 (0.94–1.77)	1.36 (1.00–2.71)
LDL, mmol/L	2.13 (1.60–2.77)	2.31 (1.70–2.90)	2.06 (1.51–2.71)
HDL, mmol/L	0.89 (0.70–1.09)	1.01 (0.80–1.28)	0.86 (0.68–1.02)
Creatinine, μmol/L	78 (63–106)	71 (59–87)	82 (65–120)
Blood urea nitrogen, mmol/L	6.0 (4.4–9.1)	4.8 (3.7–6.2)	6.9 (4.9–10.7)
eGFR, mL/min	81.5 (55.8–94.8)	89.9 (75.3–101.3)	75.2 (47.7–91.5)
Sodium, mmol/L	138.5 (135.5–140.9)	139.6 (137.0–141.6)	138.0 (135.0–140.6)
Potassium, mmol/L	4.15 (3.81–4.51)	4.19 (3.83–4.52)	4.13 (3.79–4.51)
Calcium, mmol/L	2.15 (2.06–2.24)	2.16 (2.07–2.24)	2.14 (2.05–2.23)
hs-CRP, mg/L	30.6 (5.7–78.1)	14.1 (2.2–59.6)	35.9 (9.1–85.1)
Coagulation function			
APTT	37.1 (33.5–41.3)	38.9 (35.9–43.0)	36.1 (32.7–40.4)
Prothrombin time, s	13.6 (12.8–14.4)	13.9 (13.2–14.5)	13.4 (12.5–14.3)
Thrombin time, s	17.2 (16.0–18.6)	16.5 (15.6–17.5)	17.7 (16.3–18.9)
International normalized ratio	1.05 (0.98–1.13)	1.06 (1.00–1.12)	1.04 (0.97–1.13)
Fibrinogen, mg/L	4.43 (3.55–5.56)	4.65 (3.64–5.92)	4.35 (3.51–5.43)
D-dimer, mg/L	0.98 (0.49–2.17)	0.73 (0.34–1.77)	1.08 (0.57–2.31)
Cardiac function related indicators			
NT-proBNP, pg/mL	274 (88–1037)	123 (39–384)	391 (128–1433)
NT-proBNP ratio	0.26 (0.09–0.89)	0.13 (0.05–0.38)	0.35 (0.12–1.22)
Diabetes related index			
Glucose, mmol/L	6.60 (5.40–8.86)	6.33 (5.26–8.19)	6.73 (5.50–9.28)
HbA1c, %	6.6 (6.0–7.8)	6.5 (5.9–7.4)	6.7 (6.1–8.0)
Death in hospital, %	376 (12.9)	87 (9.9)	289 (14.2)

*CKD* chronic kidney disease, *LDL* low-density lipoprotein, *HDL* high-density lipoprotein, *hs-CRP* high sensitive C-reactive protein, *eGFR* estimated glomerular filtration rate, *APTT* activate partial thromboplastin time, *HbA1c* glycosylated hemoglobin A1c.

During the period from December 1, 2022, to February 28, 2023, Tongji Hospital recorded a total of 4205 adult Chinese patients who received a diagnosis of COVID-19 (Fig. 1). NT-proBNP, NLR, and hsCRP measurements were performed on 2041 patients, accounting for 48.5% of the total study population. The initial assessment revealed the following median values: NT-proBNP (391 pg/mL, IQR 128–1433), NT-proBNP ratio (0.35, IQR 0.12–1.22), NLR (5.72, IQR 2.94–12.63), and hsCRP (35.9 mg/L, IQR 9.1–85.1). (Fig. 1, Table 1).

In comparison to the 2020 cohort, patients with COVID-19 in 2022 had a higher proportion of males (62% in 2022 vs. 51.1% in 2020, *P* < 0.001), were older (70 in 2022 vs. 63 in 2020, *P* < 0.001), and had shorter hospital stays (10 in 2022 vs. 20 in 2020, *P* < 0.001). The 2020 cohort also had a lower prevalence of hypertension (30.9% vs. 36.8%, *P* = 0.002), diabetes (14.4% vs. 20.4%, *P* < 0.001), coronary artery disease (7.3% vs. 14.5%, *P* < 0.001), chronic kidney disease (0.8% vs. 18.1%, *P* < 0.001), and tumors (2.2% vs. 12.4%, *P* < 0.001). In addition, COVID-19 patients in 2022 had a higher proportion of severe (22.3% vs. 18.6%) and critical (4.4% vs 3.4%) patients (Table 1).

Upon admission, the 2020 cohort exhibited higher systolic and diastolic blood pressure, respiratory rate, and pulse rate, as well as lower initial neutrophil and white blood cell counts, higher initial lymphocyte counts, and lower levels of D-dimer (Table 1). Indicators related to liver and renal function showed significant differences between the 2020 and 2022 subgroups, except for lactate dehydrogenase. The concentration of alanine aminotransferase was found to be higher in 2020 when compared to 2022. Conversely, the concentration of aspartate aminotransferase was observed to be higher in 2022 compared to 2020. However, the renal function of patients in 2022 was significantly worse compared to patients in 2020. In 2022, there were noticeable differences in the patients' renal function compared to 2020. Specifically, levels of blood urea nitrogen and creatinine were higher in 2022, while estimated glomerular filtration rate (eGFR) was lower. Additionally, concentrations of D-dimer were higher in 2022 compared to 2020 (Table 1).

### Clinical outcomes during hospitalization

**Figure 2:**
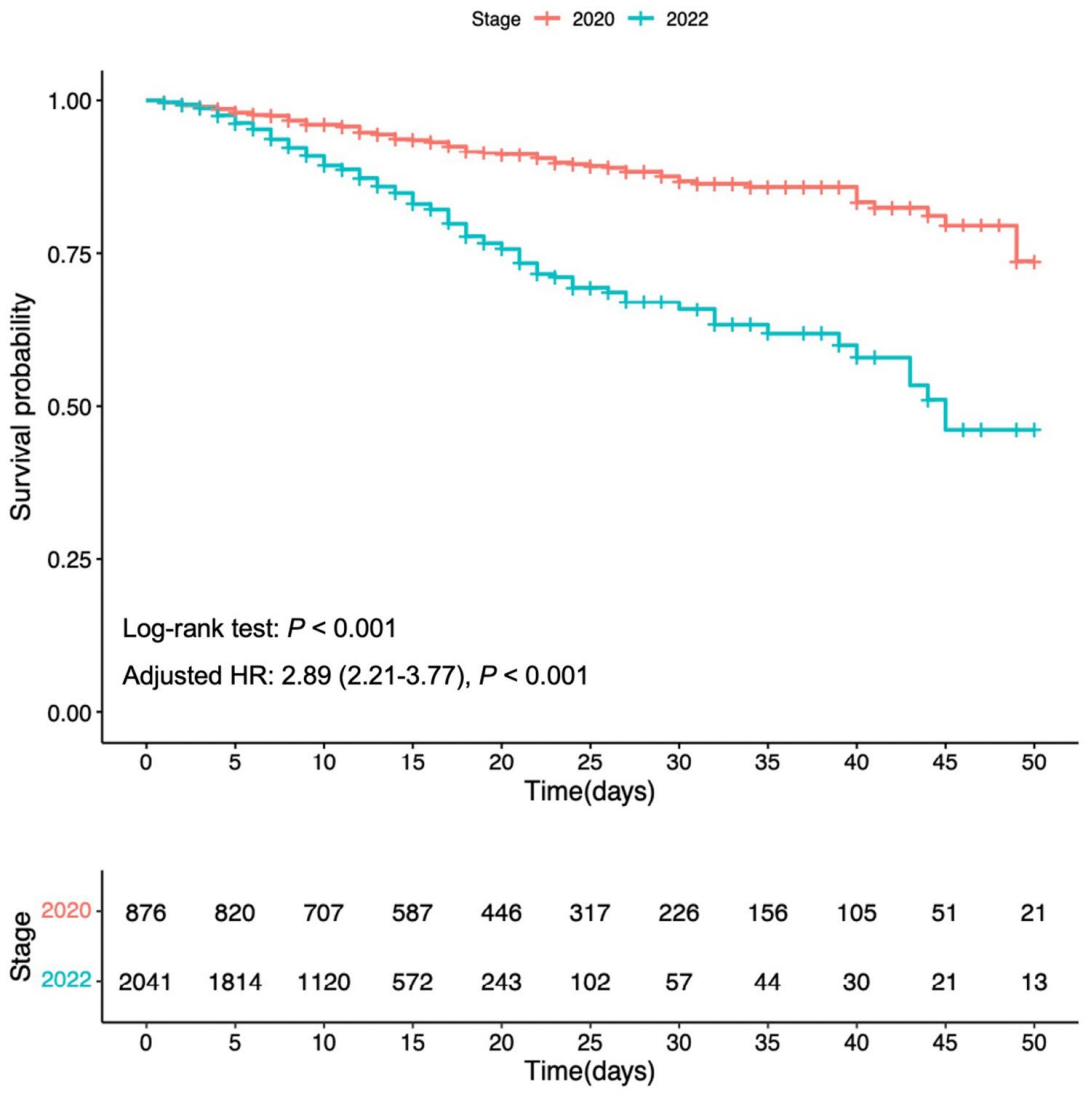
The Kaplan–Meier survival curve of enrolled COVID-19 patients in 2020 and 2022.

In this study, a total of 376 individuals (12.9%) died while hospitalized, including 87 (9.9%) deaths in 2020 and 289 (14.2%) in 2022 (*P* = 0.002) (Fig. 1, Table 1). Cumulative survival curves indicated a higher in-hospital mortality rate among COVID-19 patients in 2022 compared to 2020 (Fig. 2).

**Figure 3:**
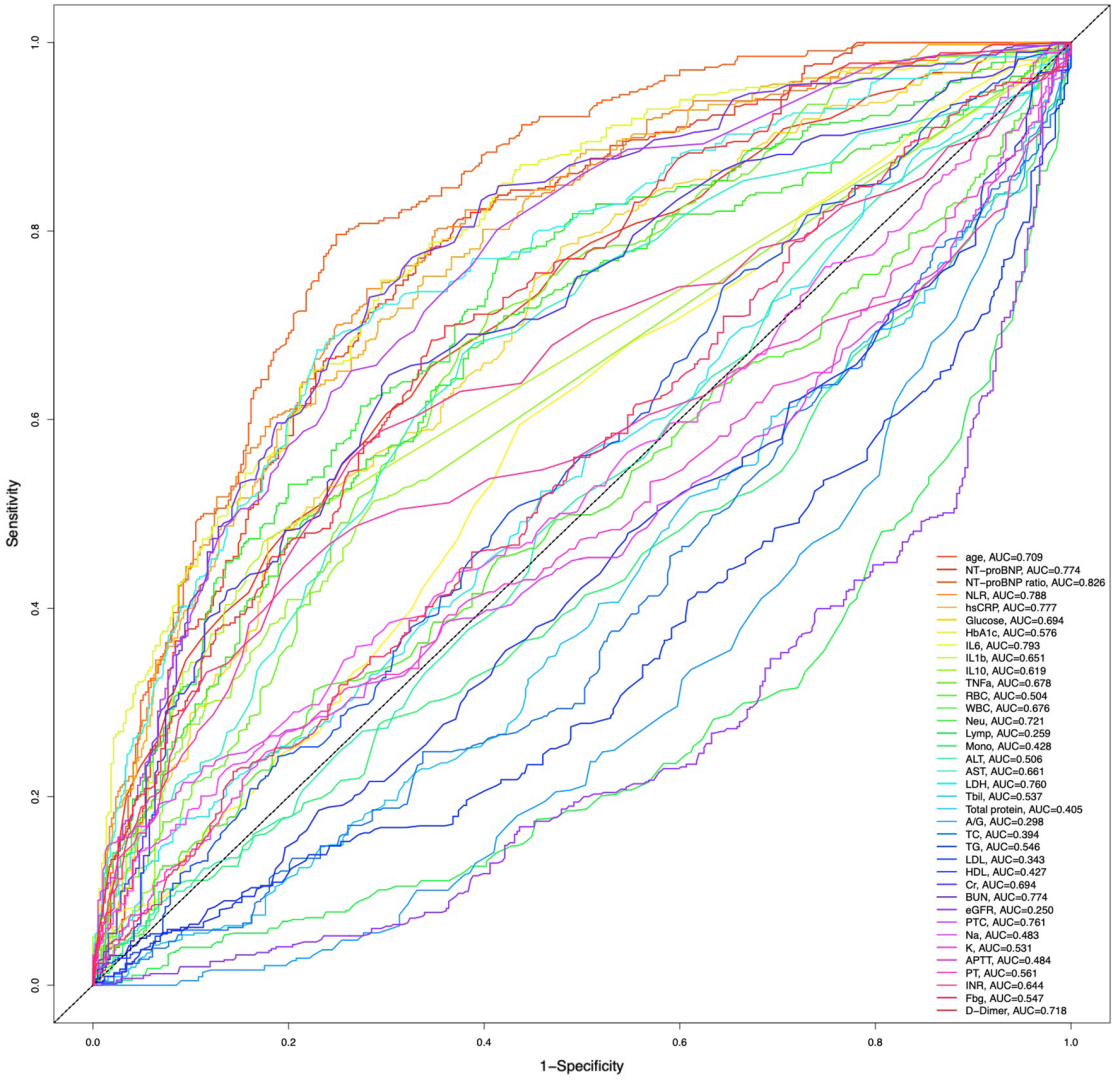
The NT-proBNP ratio and all the other clinical indicators for in-hospital death of total COVID-19 patients by time-dependent receiver operating characteristic (ROC) curves.

Among the entire participant group (Fig. 3) as well as the 2020/2022 subgroup (Fig. S1), the initial NT-proBNP ratio exhibited the largest area under the curve (AUC) in the time-dependent receiver operating characteristic (ROC) curve, indicating its potential predictive ability for in-hospital mortality (AUC = 0.826, *P* < 0.001). Additionally, hsCRP was shown to be a more reliable potential factor than NLR in 2020, whereas NLR surpassed hsCRP in predictive value in 2022 (Fig. S1).

### NT-proBNP ratio and in-hospital mortality

**Figure 4:**
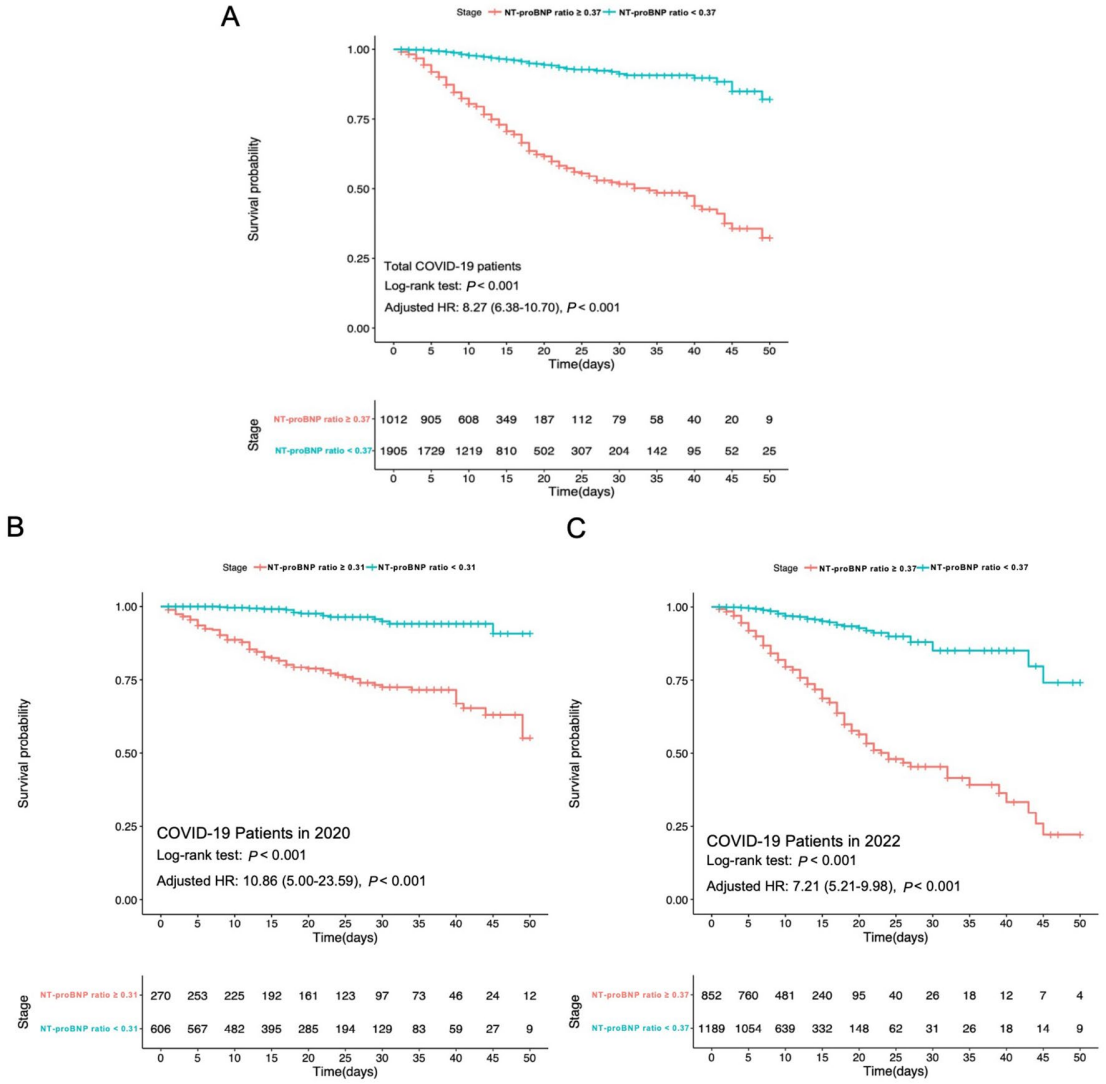
The Kaplan–Meier survival curve of enrolled COVID-19 patients with high and low NT-proBNP ratio in the total participants (**A**), in 2020 subgroup (**B**) and in 2022 subgroup (**C**).

Gender, age, hypertension, diabetes, white cell count and lactate dehydrogenase were adjusted for in hazard ratio estimates based on the results of univariate Cox regression. Both the Kaplan–Meier survival curve and proportional Cox regression analysis revealed a strong correlation between elevated NT-proBNP ratios (≥ cutoff value 0.37) and higher mortality rates (log-rank *P* < 0.001; adjusted hazard ratio [HR]: 8.27; 95% confidence interval [CI] 6.38–10.70, *P* < 0.001) among all patients, as well as within the subgroups of 2020 and 2022 (cutoff value 0.31; log-rank *P* < 0.001; adjusted HR: 10.86; 95% CI 5.00–23.59, *P* < 0.001 vs. cutoff value 0.37; log-rank *P* < 0.001; adjusted HR: 7.21; 95% CI 5.21–9.98, *P* < 0.001) (Fig. 4).

Additionally, the Kaplan–Meier survival curve and multivariate Cox model analysis revealed a significant increase in all-cause mortality associated with higher concentrations of NT-proBNP (cutoff value ≥ 592 pg/ml, log-rank *P* < 0.001; adjusted HR: 6.70; 95% CI 5.11–8.79, *P* < 0.001), NLR (cutoff value ≥ 7.29, log-rank *P* < 0.001; adjusted HR: 6.40; 95% CI 5.01–8.17, *P* < 0.001), and hsCRP (cutoff value ≥ 69.9 mg/L, log-rank *P* < 0.001; adjusted HR: 5.29; 95% CI 4.24–6.58, *P* < 0.001), as well as in the older age group (cutoff value ≥ 73 years, log-rank *P* < 0.001; adjusted HR: 2.79; 95% CI 2.25–3.50, *P* < 0.001) within the overall COVID-19 group (Fig. S2). These consistent results were also observed in subgroup analyses, with lower cutoff values in 2020 compared to 2022 for NT-proBNP (283 pg/ml vs. 593 pg/ml), NLR (3.83 vs. 8.37), hsCRP (32.5 mg/L vs. 70.4 mg/L), and age (58 years vs. 73 years) (Figs. S3, S4).

### NT-proBNP ratio risk stratification

**Figure 5:**
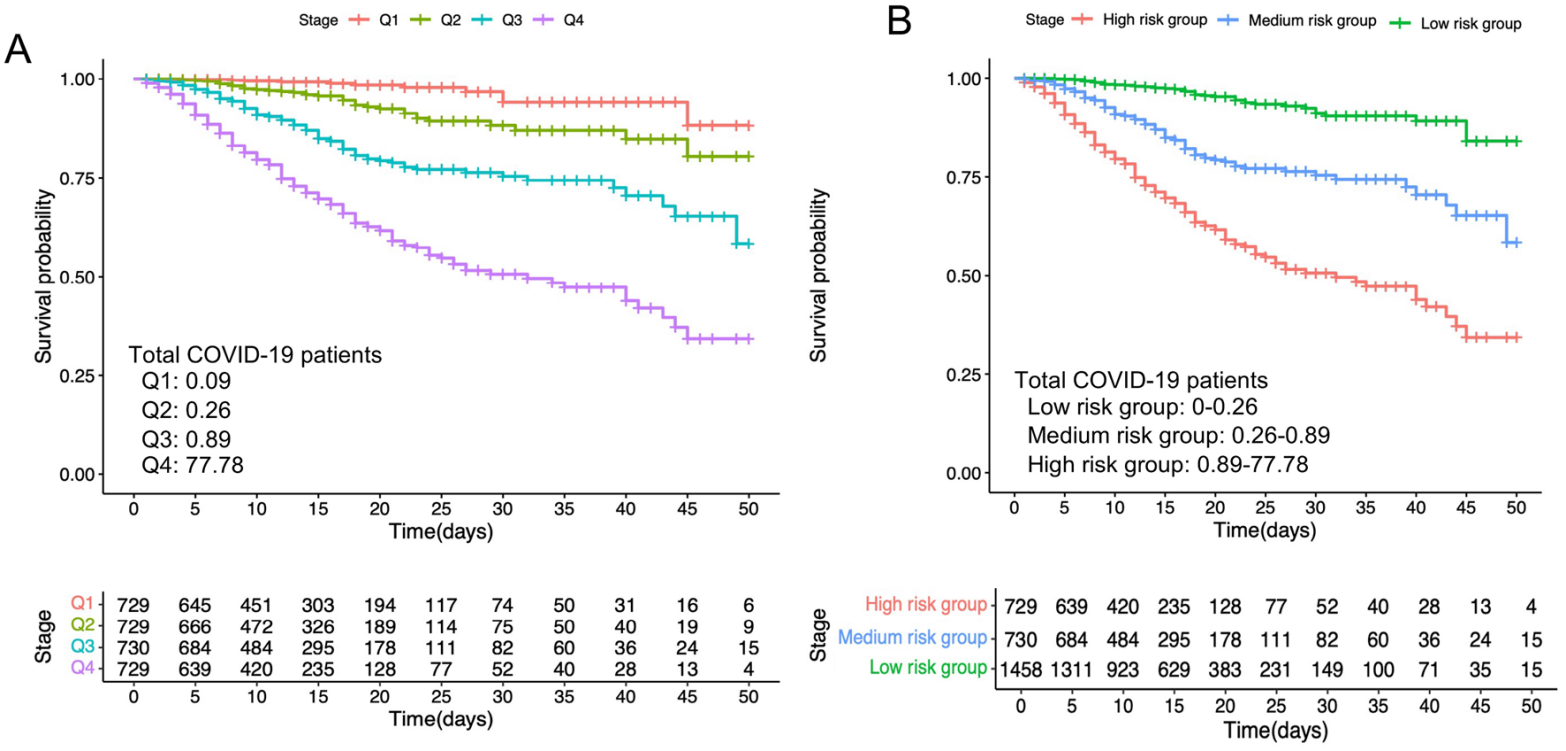
(**A**) The Kaplan–Meier survival curve of total COVID-19 patients with quartiles of NT-proBNP ratio. (**B**) Risk stratification of in-hospital mortality according to NT-proBNP ratio among total COVID-19 participants.

To assess the risk stratification of in-hospital mortality among COVID-19 participants, we categorized 2917 patients into quartiles based on the ascending order of the NT-proBNP ratio (Fig. 5). Univariable and multivariable Cox regression analyses of the entire COVID-19 patient cohort revealed that quartiles 2, 3, and 4 had a significantly higher risk of death compared to the first quartile (lowest) (Table 2).

**Table 2:** Univariate and multivariate Cox regression of NT-proBNP ratio in the total patients and different subgroups.

Subgroup		Univariable Cox regression			Multivariable Cox regression		
		HR (95% CI)	SE	*P*	HR (95% CI)	SE	*P*
Total COVID-19 patients	Quantile 2 vs quantile 1	3.38 (1.73–6.60)	0.34	< 0.001	3.14 (1.60–6.16)	0.34	0.001
	Quantile 3 vs quantile 1	9.39 (5.05–17.48)	0.32	< 0.001	8.82 (4.72–16.49)	0.32	< 0.001
	Quantile 4 vs quantile 1	22.53 (12.29–41.31)	0.31	< 0.001	22.83 (12.39–42.05)	0.31	< 0.001
COVID-19 patients in 2020	Quantile 2 vs quantile 1	5.03 (0.62–41.00)	1.07	0.131	4.82 (0.58–40.00)	1.08	0.145
	Quantile 3 vs quantile 1	8.77 (1.15–66.65)	1.04	0.035	6.91 (0.88–54.51)	1.05	0.044
	Quantile 4 vs quantile 1	44.55 (6.17–321.73)	1.009	< 0.001	45.76 (6.32–331.64)	1.01	< 0.001
COVID-19 patients in 2022	Quantile 2 vs quantile 1	4.41 (1.94–10.01)	0.42	< 0.001	4.04 (1.77–9.23)	0.42	0.001
	Quantile 3 vs quantile 1	13.44 (6.24–28.92)	0.39	< 0.001	12.06 (5.58–26.09)	0.39	< 0.001
	Quantile 4 vs quantile 1	21.30 (9.98–45.44)	0.39	< 0.001	22.22 (10.31–47.91)	0.39	< 0.001

Using a cutoff value of 0.37, we defined the low-risk group as NT-proBNP ratio < 0.37, the medium-risk group as 0.37 < NT-proBNP ratio < 0.89, and the high-risk group as 0.89 < NT-proBNP ratio < 77.78 (Fig. 5). There were no significant differences in the cutoff value of the NT-proBNP ratio and quartiles among the total patients and the two subgroups. The value in 2022 was the same as that of the total patients, slightly higher than that of 2020. Therefore, in the 2020 group, we defined the low-risk group as NT-proBNP ratio < 0.31, the medium-risk group as 0.31 < NT-proBNP ratio < 0.38, and the high-risk group as 0.38 < NT-proBNP ratio < 77.78. In the 2022 group, we defined the low-risk group as NT-proBNP ratio < 0.37, the medium-risk group as 0.37 < NT-proBNP ratio < 1.22, and the high-risk group as 1.22 < NT-proBNP ratio < 76.61 (Fig. S5).

## Discussion

This study highlights the potential of the NT-proBNP ratio as a tool in predicting in-hospital mortality among adult COVID-19 patients. The elevation of the NT-proBNP ratio upon admission may serve as an early warning sign for clinicians to identify high-risk individuals who require closer monitoring and immediate intervention.

NT-proBNP, which reflects hemodynamic stress, has shown its value in risk stratification for heart failure (HF) and other conditions like pulmonary embolism and pneumonia following SARS-CoV-2 infection^4,7^. Elevated NT-proBNP levels have been independently associated with COVID-19 mortality, even after considering factors such as chronic or acute HF^4,8^. Several studies have suggested a potential link between NT-proBNP and worse outcomes in COVID-19 patients, supporting its use for risk stratification^9–11^.

Our research demonstrated that the AUC for NT-proBNP was smaller than that of NLR or hsCRP in all three groups. However, the NT-proBNP ratio had the highest AUC in the time-dependent ROC curve and showed the highest adjusted hazard ratio in multivariate Cox regression analysis. Although NT-proBNP is known to be influenced by renal function, and we observed worse renal function in the 2022 patient cohort, our findings demonstrate that the NT-proBNP ratio emerged as a potential factor compared to NT-proBNP/eGFR (AUC = 0.757, *P* < 0.001) for predicting in-hospital mortality in COVID-19 patients.

The cytokine storm, characterized by uncontrolled and exaggerated immune responses, can result in diffuse alveolar damage, multi-organ failure, and ultimately, death in COVID-19^12^. Elevated CRP levels can signify a secondary "cytokine storm" induced by secondary bacterial pneumonia, which can independently contribute to multiorgan damage^13^. Initial elevation in CRP levels has been shown to be an independent indicator of critical illness in COVID-19 patients (AUC 0.783, *P* < 0.001)^14^. Additionally, in a time-to-mortality Cox regression analysis, a threshold cutoff of CRP ≥ 40 mg/L demonstrated good performance in predicting COVID-19 mortality^15^. These findings highlight the significance of CRP as a biomarker in assessing disease severity and prognosis in COVID-19. Our research findings also indicated that CRP has shown promising predictive performance (cutoff value = 69.9 mg/L, adjusted HR = 5.29, *P* < 0.001) for in-hospital mortality among all COVID-19 patients (AUC = 0.777, *P* < 0.001).

Researchers identified a common immune pattern in the peripheral blood of 63 hospitalized COVID-19 patients, regardless of their diverse backgrounds, showing significant changes in T cell characteristics and specific increases in cytokines/chemokines^16^. This immune signature enables early patient identification and risk-based categorization^16^. In COVID-19 patients, a higher NLR has been linked to clinical deterioration and increased mortality^17,18^. In our study, the AUC for NLR (with a cutoff value of 7.29) in predicting mortality among the total 2,917 COVID-19 patients was 0.788 (0.768 in 2020 with a cutoff value of 3.83, and 0.780 in 2022 with a cutoff value of 8.37). The previous study reported varying AUC values for mortality prediction in COVID-19 patients using NLR: 0.8126^19^, 0.7127^20^, 0.8628^21^, 0.8529^22^, 0.8130^23^, 0.9531^24^, and 0.9232^25^. However, the number of participants in each study was significantly smaller compared to ours, with the largest study having only 1004 participants. The study with the highest enrollment size also had the highest AUC value and a cutoff value of 11.8^24^. Four studies were conducted in China, while the remaining three took place in America^20^, Turkey^22^, and Pakistan^23^. Except for one study from America involving 125 patients, which had a lower AUC than ours, the rest demonstrated higher AUC values. These variations highlight the heterogeneity in findings across different studies. Overall, our results support the importance of NLR as a potential indicator for COVID-19 mortality, with our study demonstrating favorable AUC values and cutoff points compared to previous research.

In 2020, at the onset of the pandemic outbreak, there was limited understanding of the disease, leading to widespread fear and a surge of infected patients seeking hospitalization. Our results indicate a higher incidence of concomitant pathologies, such as secondary bacterial pneumonia in the 2020 group. However, in 2022, with a subsequent wave of infections, the public's response changed. The fear subsided, and people became more aware of how to handle the virus. Additionally, as the virulence of SARS-CoV-2 decreased, the majority of individuals experienced milder symptoms compared to their initial infection. As a result, except for patients with comorbidities, most individuals chose not to undergo hospital treatment. In the 2022 group, a greater proportion of patients with more comorbidities exhibited lymphocyte depletion. During the initial outbreak in 2019, a greater number of patients succumbed to SARS-CoV-2 infection at an early stage or displayed signs of interstitial pneumonia in chest computed tomography (CT) before viral nucleic acid detection. In contrast, in 2022, fewer patients died at an early stage or showed signs of chest CT abnormalities, and if abnormalities were present, they appeared after viral nucleic acid detection. In addition, due to the promotion of China's national COVID-19 vaccination policy, the entire population has been vaccinated once or more times in and after 2021^26^. All COVID-19 patients enrolled in 2022 have received the vaccination. However, in the research cohort, none of the COVID-19 patients in 2020 received the vaccination because of the lack of effective vaccines in the emergency situation.

Furthermore, we observed that the concentration of D-dimer was higher in the 2022 patients compared to those in 2020. This observation is likely associated with thrombo-inflammation, a process implicated in adverse events related to COVID-19. Thromboinflammation is characterized by dysregulation of endothelial antithrombotic function in response to inflammatory stress, resulting in leukocyte recruitment, complement and platelet activation, and enhanced microvascular coagulation^27,28^. These findings highlight the evolving nature of the disease and the involvement of different pathologies and mechanisms over time.

## Limitations

It is important to address the limitations of our study, which was a single-center, retrospective, observational study. The lack of consistent evaluation of NT-proBNP concentration at hospital admission in all patients in 2020 may introduce selection bias and underestimate mortality in the 2020 subgroup. In the second epidemic, patients were arranged to be hospitalized in various departments of hospitals due to medical squeeze, and doctors in different departments also had different understanding of the impact of the novel coronavirus on other systems. The detection rate of NT-proBNP is not high. However, for inclusion in the study, only those patients with detectable NT-proBNP values were enrolled. In addition, during the initial outbreak of the pandemic in 2020, there was a rapid and substantial increase in the number of patients, exceeding the capacity of laboratory to confirm SARS-CoV-2, and cases clinical diagnosed based on imaging evidence of pneumonia, along with relevant epidemiological background and clinical symptoms were also included. While our findings may not fully represent the entire COVID-19 population, they provide essential insights for prognosticating patients infected with the continuously mutating SARS-CoV-2 virus.

## Conclusion

Based on our study findings, we propose that the NT-proBNP ratio serves as a potential indicator for adult COVID-19 patients. These results contribute to our understanding of risk stratification and provide valuable information for clinical decision-making in managing COVID-19 patients. However, further research and validation studies are needed to confirm and expand upon these findings.

### Supplementary Information

Supplementary Figures.

## Data Availability

The data underlying this article will be shared upon reasonable request by the corresponding author.

## Abbreviations

SARS-CoV-2: Severe acute respiratory syndrome coronavirus 2
COVID-19: Corona virus disease 2019
NT-proBNP: N-terminal pro B-type natriuretic peptide
HF: Heart failure
CKD: Chronic kidney disease
LDL: Low-density lipoprotein
HDL: High-density lipoprotein
NLR: Neutrophil-to-lymphocyte ratio
hs-CRP: High sensitive C-reactive protein
eGFR: Estimated glomerular filtration rate
APTT: Activate partial thromboplastin time
HbA1c: Glycosylated hemoglobin A1c
