# Supplementary material for: NT-proBNP ratio is a potential predictor for COVID-19 outcomes in adult Chinese patients: a retrospective study
Source: Sci Rep. 2024 Mar 11;14:5906. doi: 10.1038/s41598-024-56329-2 (PMC10928211; doi:10.1038/s41598-024-56329-2)

Figure S1. The NT-proBNP, NT-proBNP ratio, age, NLR and hsCRP for in-hospital death of COVID-19 patients in 2020 (A) and 2022 (B) by receiver operating characteristic (ROC) curves.

Figure S2. The Kaplan–Meier survival curve of total enrolled COVID-19 patients with high and low NT-proBNP (A), age (B), NLR (C) and hsCRP (D).

Figure S3. The Kaplan–Meier survival curve of enrolled COVID-19 patients in 2020 with high and low NT-proBNP (A), age (B), NLR (C) and hsCRP (D).

Figure S4. The Kaplan–Meier survival curve of enrolled COVID-19 patients in 2022 with high and low NT-proBNP (A), age (B), NLR (C) and hsCRP (D).

Figure S5. The Kaplan–Meier survival curve of COVID-19 patients with different levels of NT-proBNP ratio in 2020 (A&B) and 2022 (C&D)

Supplementary Figure 1

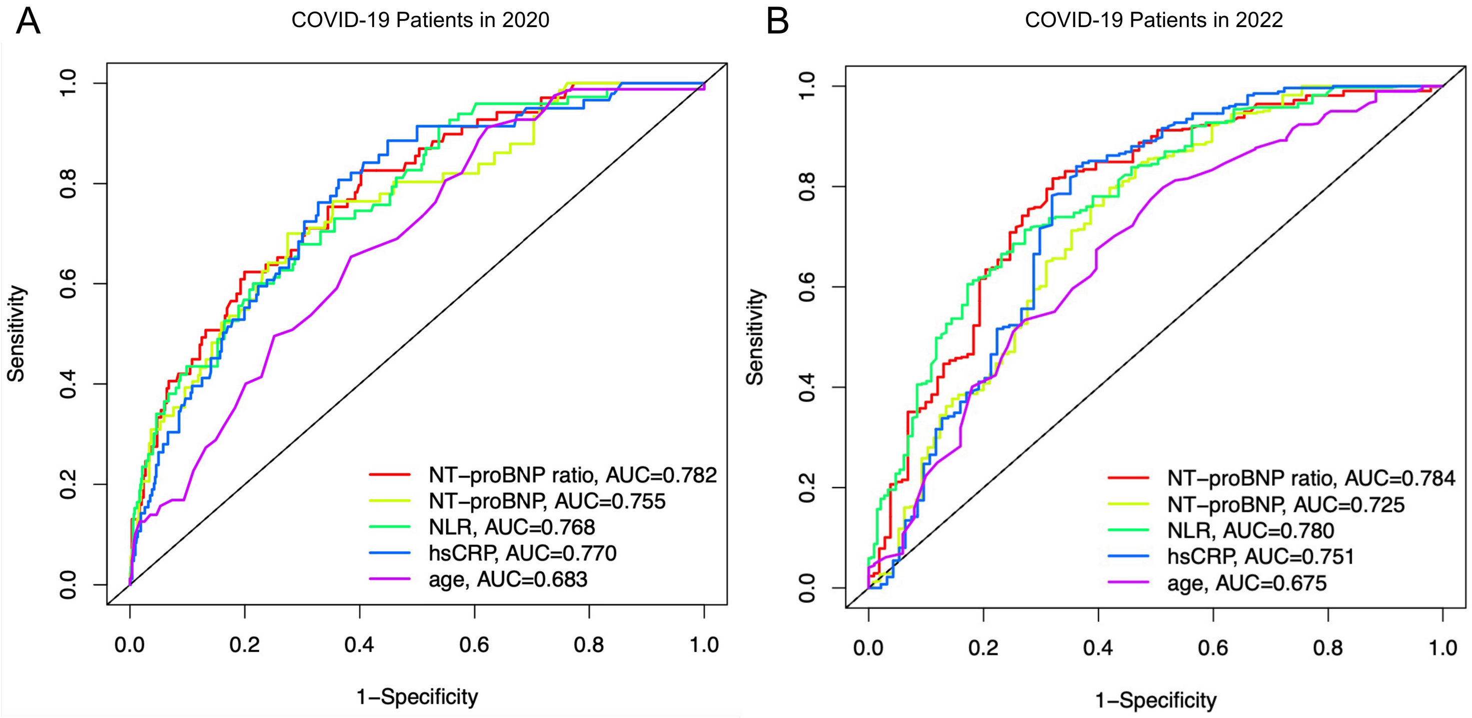

Supplementary Figure 2

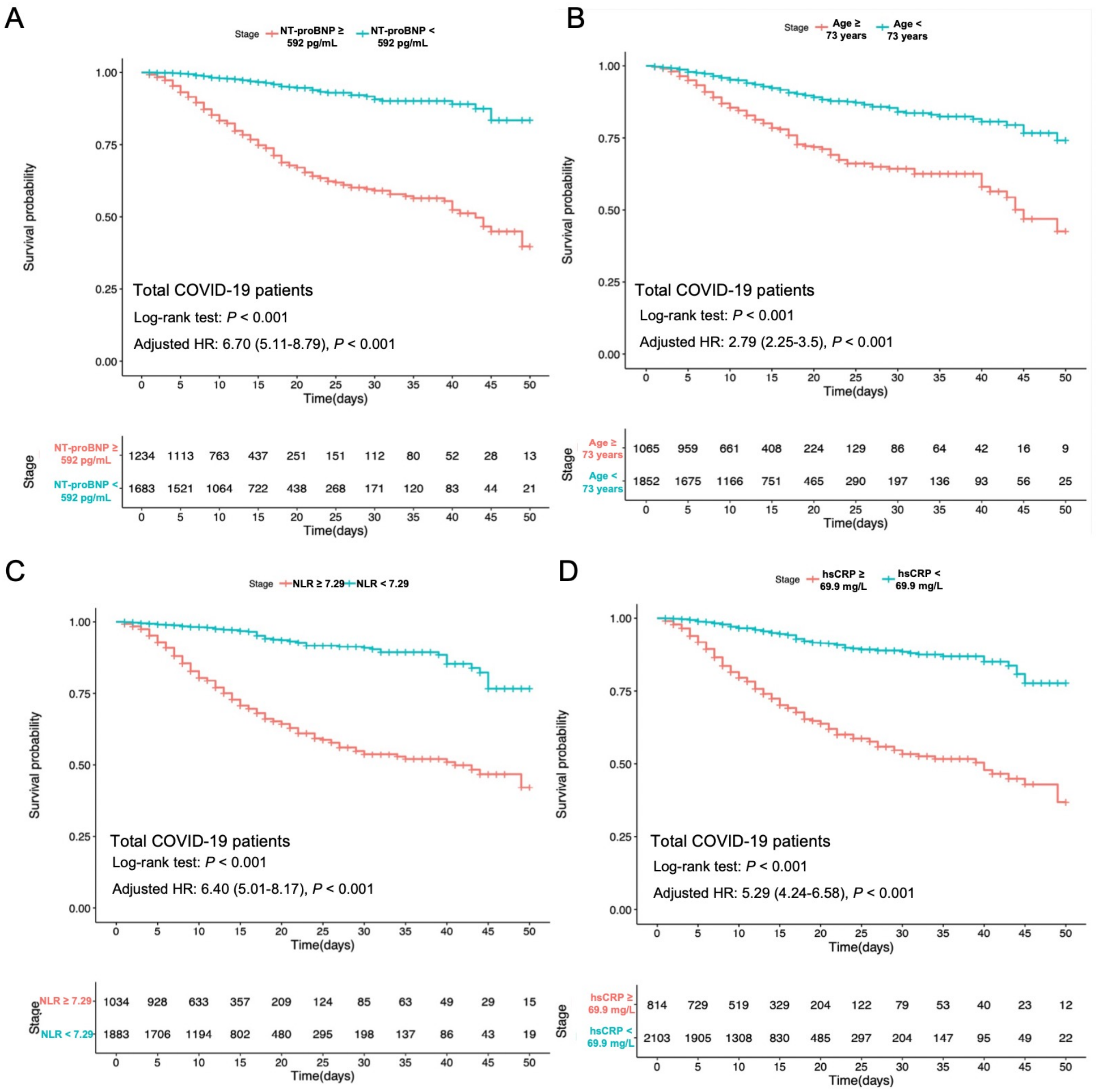

Supplementary Figure 3

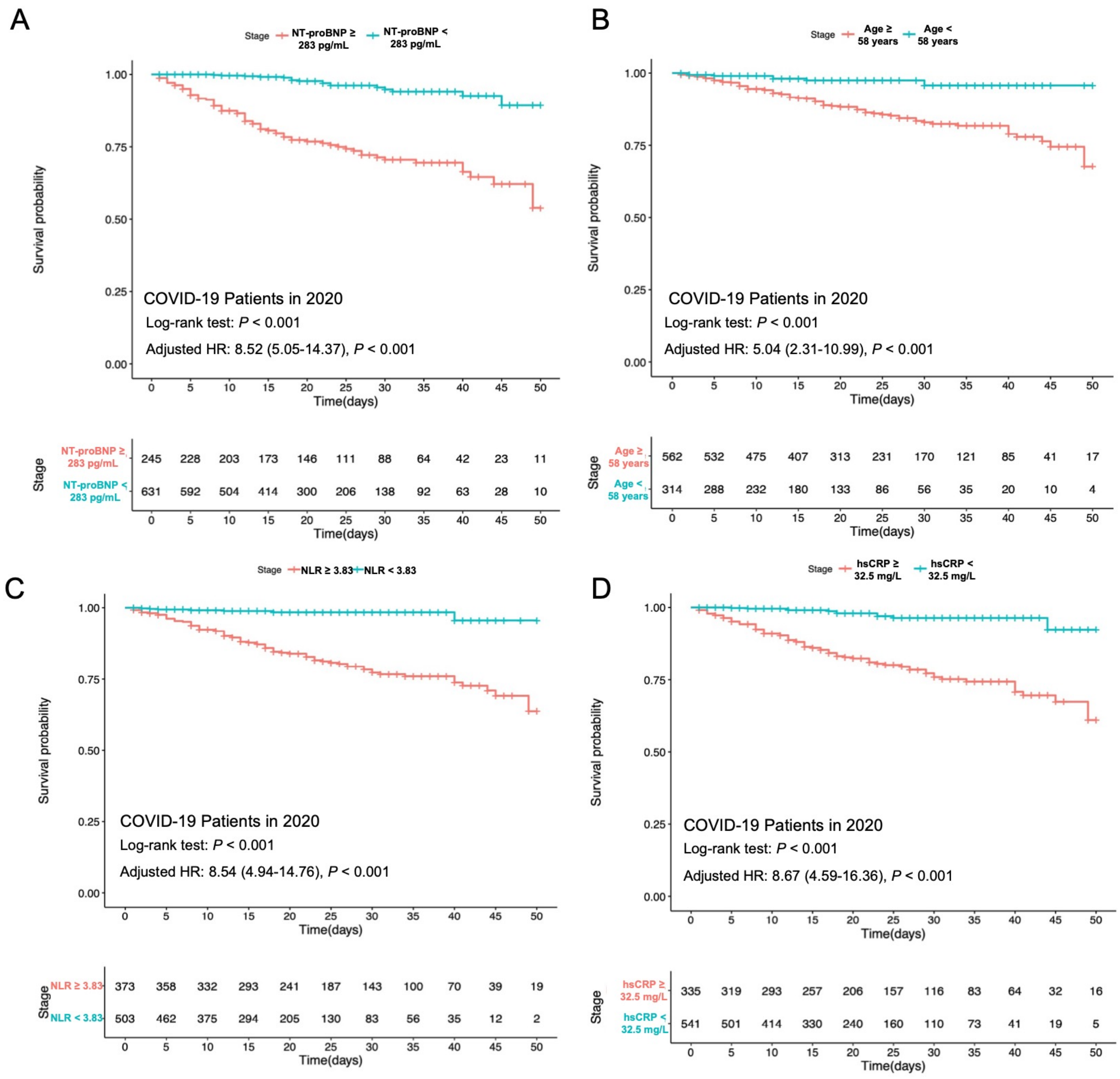

Supplementary Figure 4

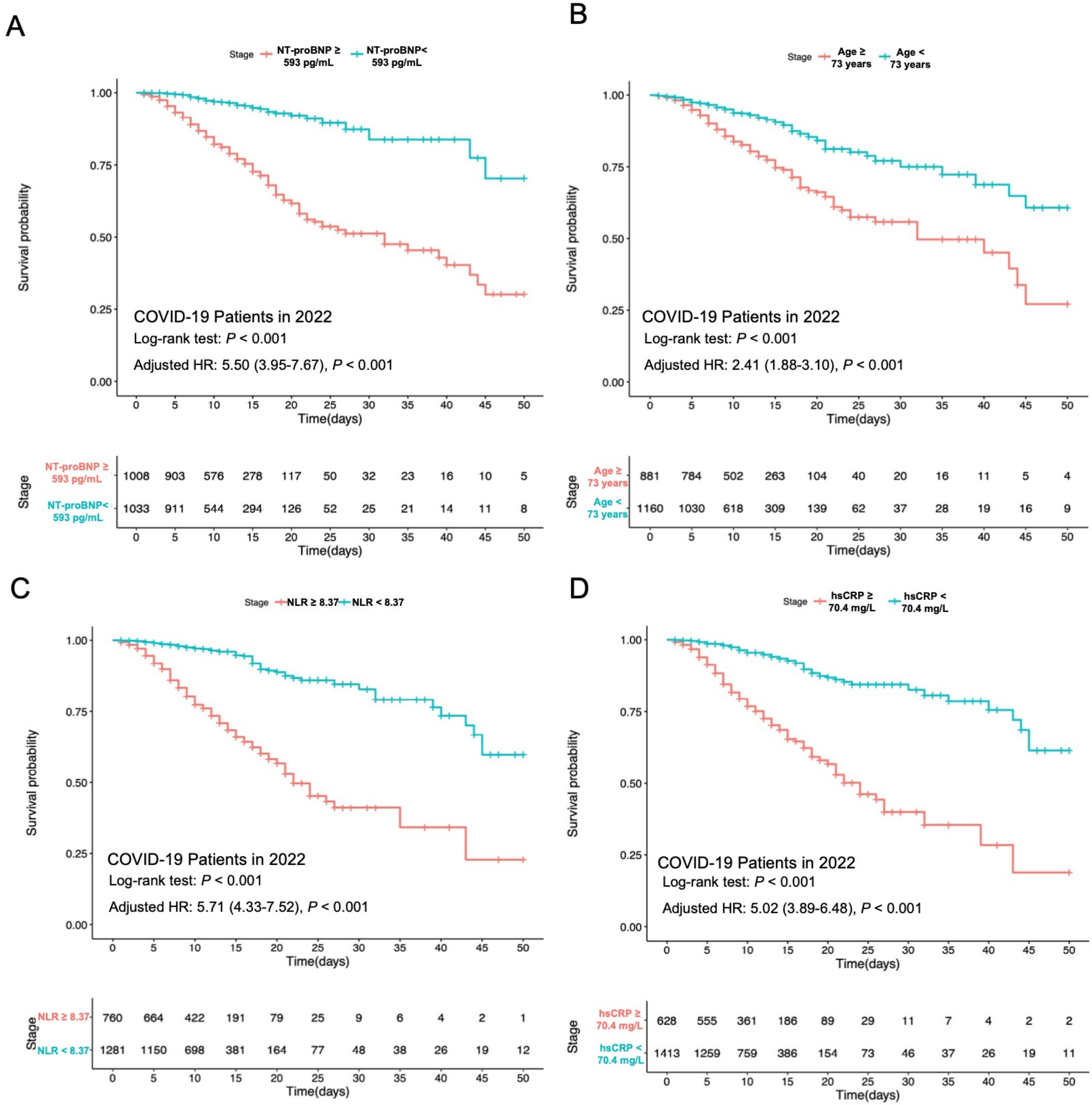

Supplementary Figure 5

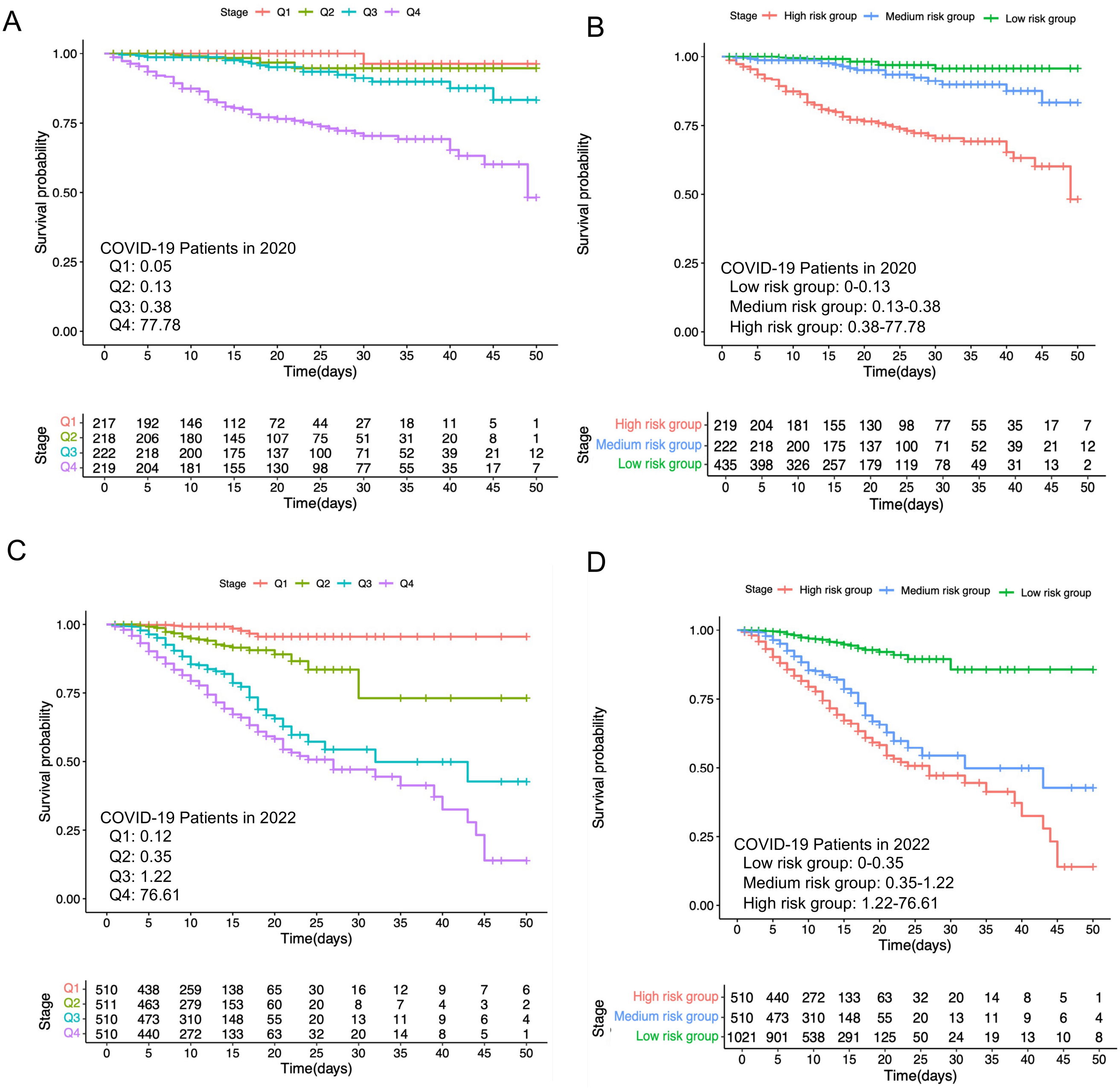

Supplement: Supplementary file 1 — Supplementary Figures. [file 41598_2024_56329_MOESM1_ESM.pdf]
